# Evaluation and Classification of Mast Cell Disorders: A Difficult to Manage Pathology in Clinical Practice

**DOI:** 10.7759/cureus.22177

**Published:** 2022-02-13

**Authors:** Polliana Mihaela Leru

**Affiliations:** 1 Clinical Department 5, Carol Davila University of Medicine and Pharmacy, Bucharest, ROU; 2 Internal Medicine, Colentina Clinical Hospital/Carol Davila University of Medicine and Pharmacy, Bucharest, ROU

**Keywords:** serum tryptase, rare diseases, mastocytosis, mast cell activation syndrome, mast cells

## Abstract

Mast cells are granulocytic immunomodulatory cells with an important role in physiologic and pathogenic processes due to their location at the junction between the internal and external environment and to their capacity to release a broad range of active mediators. Mast cells mediators have both pro-inflammatory and anti-inflammatory activities and are implicated in various and complex pathology. Mast cells disorders (MCDs) represent a heterogeneous pathology, with frequently difficult and challenging evaluation and diagnostic workup. MCDs can be primary, secondary to other diseases, or idiopathic. Increased research interest in this field was noted during the last decade and various classification criteria, as well as diagnostic and treatment recommendations, were proposed. The aim of this paper is to review the most recent published data on the classification and evaluation of mast cells disorders and to point out the main difficulties in diagnosing and managing these complex diseases in medical practice.

## Introduction and background

Mast cells (MCs) are complex and interesting cells, even after 140 years from their discovery, by Paul Ehrlich, who is considered the founder of modern immunology [1]. Despite being known for decades to play a mainly proinflammatory role in allergic reactions, it is now accepted that MCs are more complex players in many physiological and pathological processes [2]. Recent data showed that MCs are functionally and phenotypically heterogeneous, influenced by the microenvironment where they mature. Besides their major implication in immunoglobulin E (IgE)-mediated allergic diseases, they are recognized as important in host defense, innate and acquired immunity, homeostasis, and immunoregulation [3]. Recent research studies confirmed the complex implication of mast cells in many human pathological conditions such as infections, tumor development, cardiovascular diseases, and systemic disorders [2]. Actual data from the literature highlight the role of mast cells in different disorders and speculate the possibility that distinct subpopulations of mast cells, with at least two major subtypes, M1 and M2, could play different or even opposite roles in various pathological conditions [4]. Mast cells play important immunomodulatory roles, releasing a broad range of proinflammatory as well as anti-inflammatory mediators and are strategically located at sites that interfere with the external environment such as the skin, lung, and intestine [5]. Mast cells are morphologically defined by numerous electron-dense cytoplasmic granules, which contain a plethora of active mediators, such as biogenic amines (histamine, serotonin), serines and other proteases (tryptase, serine S1, chymase, cathepsin G, granzyme, carboxypeptidase), lysosomal enzymes, and proteoglycans (heparin, chondroitin sulfates) [3].

MCs activation can be measured by monitoring the release of pre-stored granule mediators (degranulation), mostly histamine and tryptase levels, which can be an early and rapid event following cells stimulation. These pre-formed mediators induce a rapid allergic response, initiating the recruitment of leukocytes and other cells and activation of the innate immune and inflammatory processes [6-7].

MCs dysfunction or abnormal location may result in a broad range of mast cells activation disorders (MCDs), which can be primary, secondary to other pathological processes, or idiopathic [8]. Mast cell dysfunction may involve multiple organ systems, occur locally (e,g., urticaria, rhinitis), or systemically (e.g., anaphylaxis), and could be implicated in a large spectrum of diseases.

The aim of this paper is to review the most recent published data on the classification and evaluation of mast cell disorders and to point out the main difficulties in diagnosing and managing this complex group of disorders in medical practice.

## Review

Diagnosis and classification of mast-cell disorders

Primary MCDs include clonal disorders, such as mastocytosis, mast cell sarcoma, mast cell leukemia, and monoclonal mast cell activation syndrome (MMCAS), a spectrum of rare and well-defined diseases associated with clonal expansion of MCs in the skin and/or other tissues and organs (Table 1).

**Table 1:** Classification of mast cells disorders Adapted from Khokhar & Akin, 2019 [9] IgE: immunoglobulin E; MCAS: mast cell activation syndrome

MCDs type	Clinical entities
Primary	Mastocytosis (cutaneous and systemic), monoclonal mast cell activation syndrome (MMAS), mast cell sarcoma, mast cell leukemia, mastocytoma
Secondary	IgE-mediated hypersensitivity reactions, urticaria, angioedema, food and drug anaphylaxis, mast cell hyperplasia (owing to systemic diseases such as chronic infection or autoimmune disease)
Idiopathic	MCAS, idiopathic anaphylaxis, chronic idiopathic urticaria/angioedema

The most commonly involved organs in primary MCDs are the bone marrow, gastrointestinal tract, liver, spleen, and lymph nodes. These disorders are due to an intrinsic defect of the MCs or its progenitors, mainly the somatic activation mutations in the tyrosine kinase receptor (KIT) proto-oncogene, the most common being aspartic acid 816 valine mutation (D816V) [10].

The diagnosis and classification of mastocytosis are based on World Health Organization (WHO) criteria outlined in 2016 when mastocytosis was considered a distinct disease and not a subgroup of myeloproliferative neoplasms [11]. The classification was updated and refined in 2019, and other subtypes with various organ systems and bone marrow B and C findings were added (Table 2) [12].

**Table 2:** WHO classification of mastocytosis Adapted from Pardanani A, 2019 [12] *B findings are: High MCs burden on BM biopsy (>20% infiltration), serum tryptase >200 ng/ml, signs of dysplasia or myeloproliferation with normal/slightly abnormal blood counts, mild hepatosplenomegaly, and/or lymphadenopathy. **C findings are more severe signs: BM dysfunction with cytopenias, caused by MCs infiltration, hepatosplenomegaly with ascites, portal hypertension, hypersplenism, bone lesions (osteolytic) with pathological fractures, and gastrointestinal MCs infiltration leading to malabsorption and weight loss. MCs - mast cells, BM - bone marrow, SM - systemic mastocytosis, CM - cutaneous mastocytosis, AHN - associated hematological neoplasm

Clinical and histological form	Sub-type and characteristics
Cutaneous mastocytosis (CM)	Urticaria pigmentosa, diffuse cutaneous mastocytosis, solitary mastocytoma of the skin
Indolent systemic mastocytosis (ISM)	Generally low MCs burden in BM, criteria for SM are present, no C findings, maybe preceded or accompanied by CM, isolated BM mastocytosis (provisional variant)
Smoldering systemic mastocytosis (SSM)	ISM plus 2/more B findings*, no C findings**, high MCs burden
Systemic mastocytosis with an associated hematological neoplasm (SM-AHN)	Criteria for SM are present plus criteria for AHN as a distinct hematological disorder
Aggressive systemic mastocytosis (ASM)	Criteria for SM plus one/more C findings
Mast cell leukemia (MCL)	Criteria for SM are present, BM with diffuse, dense MCs infiltration >20%, immature and atypical MCs, >10 MCs in peripheral blood (<10% in aleukemic variant)
Mast cell sarcoma	Localized destructive tumor, without evidence of SM

Confirmation of systemic mastocytosis diagnosis can be done in the presence of either a major criterion plus one minor criterion or at least three minor criteria (Table 3).

**Table 3:** Diagnostic criteria for systemic mastocytosis Adapted from Valent P et al. [13] MCs - mast cells, BM - bone marrow

Major criterion
Multifocal, dense MCs infiltrates in BM or other extracutaneous organs (>15 MCs in aggregates)
Minor criteria
BM with >25% of all MC infiltrates are immature MCs or with atypical morphology
Activating cKIT mutation (D816V) in blood/BM/extracutaneous organ
Increased persistent serum tryptase >20 ng/ml (in the absence of associated myeloid neoplasm)
MCs in BM/blood/extracutaneous organ express one or more of CD25 and/or CD2 and/or CD30

Cutaneous Mastocytosis

This is the most common MCD in children, usually presented as urticaria pigmentosa, and is considered a self-limited, benign condition, due to generally favorable outcomes and spontaneous regression at puberty [14]. Clinical presentation of systemic mastocytosis (SM) in adults is very heterogeneous and the diagnosis can be more challenging in the absence of skin involvement, due to variable and multiple organ dysfunction [15]. The typical presentation of cutaneous mastocytosis in adults is urticaria pigmentosa, a maculopapular monomorphic fixed exanthema, which is associated with systemic involvement in over 90% of the cases and may precede other clinical symptoms with many years [16].

Systemic Mastocytosis

This is a rare disease, generally seen by different medical specialties, depending upon the most prominent symptoms. Due to the rarity of this disorder and lack of recognition, there is often a delayed diagnosis. Indolent systemic mastocytosis (ISM) is the most frequent form of mastocytosis in adults, representing more than 80% of mastocytosis cases. Other two forms of systemic mastocytosis include adult patients with anaphylaxis induced by Hymenoptera venom, without skin lesions, and childhood cutaneous mastocytosis progressing to adult systemic disease, without cKIT mutation [17].

**Figure 1:**
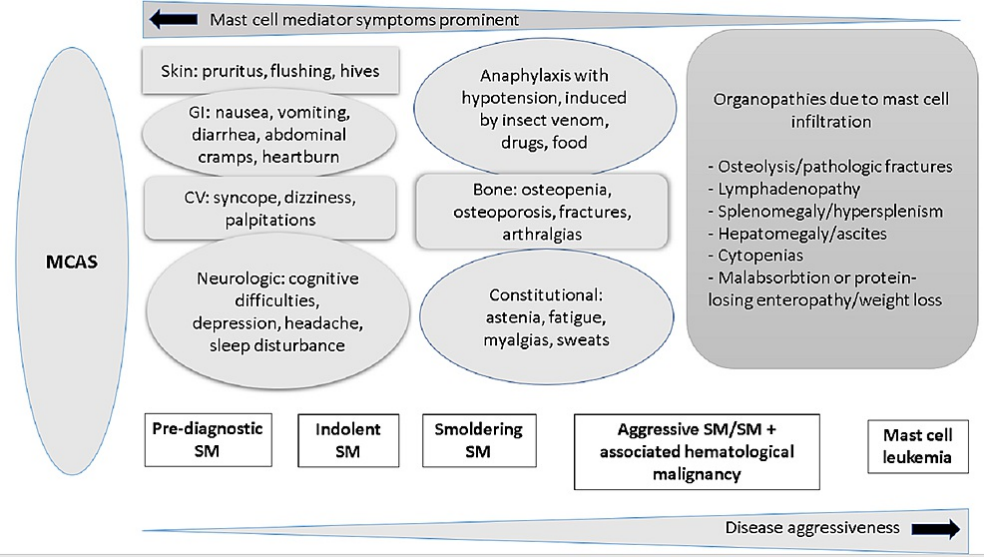
Clinical spectrum of systemic mastocytosis Adapted from Pardanani A, 2019 [12] Permission to reuse the image is obtained from John Wiley and Sons.

A high index of suspicion is required mainly in the clinical setting of unexplained anaphylaxis, osteoporosis in young patients, episodes of flushing, persistent and refractory abdominal cramping, or gastrointestinal disease (Figure 1).

The term "pre-diagnostic" systemic mastocytosis is proposed for those cases with only one to two minor criteria and abnormal MCs infiltration in the BM, which do not meet the consensus diagnostic criteria for SM [12].

Patients with mastocytosis, irrespective of disease variant, age, or laboratory tests may have mediator-induced symptoms and/or osteopathy [18]. The clinical picture can be mild, severe, distressing, or life-threatening. It is influenced by co-morbidities, the most relevant being IgE-dependent allergies, psychiatric diseases, obesity, and vitamin D deficiency [19]. Other diseases, such as concomitant infections, chronic inflammation, and food intolerances, may aggravate symptoms in patients with mastocytosis [10].

Symptoms possibly induced by mast cell mediators may determine heterogeneous clinical pictures in some patients, often attributed to presumptive intolerances or multiple food or drug allergies that require complex allergist evaluation. Drug allergies generally represent an important concern for both patients and doctors, mainly for primary care physicians who may face high pressure from this perspective, as may be the case of chronic spontaneous urticaria, unexplained recurrent angioedema, or anaphylaxis [20]. Patients with MCDs are at high risk for adverse reactions due to some medications known for their potential to induce mast cell degranulation (e.g. fluoroquinolones antibiotics, opioids, neuromuscular blocking agents, nonsteroidal antiinflammatories, etc.) and need particular caution and monitoring. Confirmation of the systemic mastocytosis diagnosis is based on expert recommendations and guidelines criteria but may be difficult in cases with incomplete diagnostic criteria, requiring allergist expertise and multidisciplinary evaluation [21].

Mast Cell Activation Syndrome (MCAS)

This is a very heterogeneous group of disorders, with unknown prevalence, characterized by episodic spontaneous mast cell activation and degranulation and nonspecific symptoms, with variable severity but without MC hyperplasia (non-clonal proliferation) [22]. Diagnosis of mast cell activation syndrome (MCAS) is very difficult in clinical practice due to symptoms variability and lack of specificity, therefore, it is generally recommended to be considered in selected patients with appropriate clinical and laboratory profiles, when other conditions have been excluded [23]. 

The most frequent symptoms reported by patients with MCAS are gastrointestinal - crampy abdominal pain, diarrhea, nausea, vomiting; cardiovascular - hypotension, tachycardia, syncope; cutaneous - pruritus, urticaria, flushing, angioedema; respiratory - wheezing, stridor [24]. Patients with MCAS may have a variable clinical phenotype, affecting multiple organ systems but the key feature is recurrent episodes of severe symptoms (anaphylaxis), with concurrent involvement of a minimum of two organ systems and association with an acute increase of specific biologic mediator levels, considered biomarkers of MC activation, mainly the serum tryptase, rarely others (histamine, leukotrienes, prostaglandins) [25]. Diagnosis criteria for MCAS have been accepted by expert consensus, based on the best evidence, and require that all three criteria are concomitantly fulfilled [26].

Consensus Diagnosis Criteria for MCAS [25]

A. Episodic and recurrent symptoms of mast cell mediator release, affecting two or more organ systems (often in the form of anaphylaxis)

B. Complete resolution of symptoms or decrease in the frequency or severity of symptoms with anti-mast cell mediator therapy (antihistamines, leukotriene modifiers, and mast cell stabilizer agents)

C. Evidence of an increase in a validated urinary or serum marker of MCAS (ideally with reproducible results obtained during more than one symptomatic episode)

Particular forms of MCDs

Idiopathic MCAS

This* *is a large and heterogeneous clinical entity that includes not only idiopathic anaphylaxis (IA) but also patients whose symptomatic episodes do not meet the definition criteria for anaphylaxis or experience episodes due to known triggers mixed with other spontaneous ones [22].

Idiopathic Anaphylaxis

This is considered a subtype of MCAS since mast cells are the only effector cells of anaphylaxis in humans and are defined when no presumed or proven cause or trigger of anaphylaxis can be identified [27].

Most authors consider that the MCAS diagnosis is attributed to patients with severe and episodic symptoms affecting many organ systems, suggesting anaphylaxis, rather than to those with nonspecific symptoms, such as recurrent flushing and/or gastrointestinal symptoms.

Isolated Bone Marrow Mastocytosis

This is a particular variant of indolent systemic mastocytosis without skin involvement, with normal or near-normal serum tryptase level, leading to a more difficult and delayed diagnosis compared to other forms. Symptoms such as unexplained anaphylaxis, flushing, osteoporosis, gastrointestinal ulcerative disease, or abdominal cramping may suggest systemic mastocytosis, after exclusion of other disorders [15].

MCDs Associated With Blood or Tissue Hypereosinophilia (HE)

This can be encountered in rare cases since mast cells and eosinophils have complex and complementary roles in many diseases, including allergies. There is an increasing research interest in both mast cells and eosinophils, the bidirectional interaction between these cells being confirmed, and their important implication in more than allergic diseases and in many inflammatory, cardiovascular, and neoplastic disorders [28]. The prognostic impact of hypereosinophilia (HE) in mastocytosis patients was evaluated and collected from the European Competence Network on Mastocytosis (ECNM) Registry, concluding that HE did not influence mediator-related symptoms or allergic reactions but was related with worse prognosis, compared with mastocytosis patients without hypereosinophilia [29].

Laboratory tools for MCDs diagnosis

The most important laboratory tools in the evaluation and confirmation of a possible MCD are serum tryptase measurement, molecular studies to detect cKIT characteristic mutation, bone marrow examination, and possibly other biopsies if indicated by the clinical picture (mainly the skin and gastrointestinal tract) [30]. Serum tryptase is a reliable blood test and should be measured and monitored in all cases with clinical suspicion of MCD and in all anaphylaxis and severe allergic cases. Tryptase is contained in the secretory granules of human MCs and is considered the marker of MC disorders since it is produced almost exclusively by MCs, excepting small amounts produced by basophils [31]. Serum tryptase level is normal or slightly elevated in patients with cutaneous mastocytosis, and this may be also normal in the early stages of systemic mastocytosis, it increases over a period of months or years and then remains stable. The upper normal value of serum tryptase is 11.4 ng/ml in most laboratories and its level usually correlates with the MCs burden [32]. Elevated serum tryptase levels are not specific for mastocytosis and can be found in other diseases: severe anaphylaxis with hypotension, hematologic myeloid malignancies, chronic end-stage renal failure, some cases of urticaria, onchocerciasis, and familial hypertryptasemia [33].

Difficulties in diagnosis and management of MCDs in clinical practice

Mast cells activation disorders and syndromes represent a difficult-to-manage group of clinical entities in medical practice, ranging from mild and persistent to acute and very severe diseases. While urticaria pigmentosa is a benign form of cutaneous mastocytosis, usually with mild discomfort and a good prognosis, idiopathic anaphylaxis is a severe disease with possible fatal outcomes. Patients who consider themselves having a mast cell disorder may be seen by many physicians before diagnosis confirmation [34]. A mast cell disorder may be suggested in cases with idiopathic anaphylaxis or a severe allergic reaction due to Hymenoptera sting or unexplained syncope. Symptoms, including chronic urticaria, uncontrolled asthma, or persistent gastrointestinal symptoms, are inconsistent with a mast cell disorder.

The diagnosis and management of mast cell disorders are difficult in clinical practice, requiring vast knowledge, a multidisciplinary approach, and personalized medicine procedures. A high index of suspicion is very important before addressing patients with possible MCDs to allergists or other specialists and the contribution of an experienced pathologist in diagnosis confirmation is very important [35]. The consensus diagnostic criteria for mastocytosis and MCAS should be considered in all patients with a high index of clinical suspicion, according to the latest guidelines and expert recommendations [13,25-36].

A multidisciplinary approach is mandatory for the optimal management of MCDs due to complex molecular mechanisms, multiorgan involvement, and variable clinical course [19]. The therapeutic strategy in non-advanced cases is based on MCs mediators-induced symptoms, control of risk factors and co-morbidities, and psychological support.

Due to remarkable progress in this area during the last decade and according to precision and personalized medicine approaches, the mast cell diseases' outcome and patients' quality of life have significantly improved and further progress can be anticipated. Recent inquiry of MCDs patient groups from 12 countries referring to patients' concerns and expectations from the scientific community revealed the need for better education and knowledge of physicians, increased awareness, better access to specialized centers, improved diagnostic criteria, and better treatments [37].

## Conclusions

Mast cell disorders are complex clinical entities, considered more frequent than previously believed, but certainly underdiagnosed. Diagnosis confirmation may be lengthy, with multiple diagnostic procedures, and may involve unpleasant diagnostic procedures and high costs. Similar to the case of other rare diseases, there is a clear need for developing more specialized centers in mast cells disorders and improving interdisciplinary collaboration and expertise in this medical area.
